# Supplementary material for: An open-source framework for large-scale, flexible evaluation of biomedical text mining systems
Source: J Biomed Discov Collab. 2008 Jan 29;3:1. doi: 10.1186/1747-5333-3-1 (PMC2276192; doi:10.1186/1747-5333-3-1)
Supplement: Additional file 1 — Evaluation platform source code. The additional file contains the software evaluation framework discussed in this paper. The most current release can be downloaded from SourceForge.net [1]. See the accompanying README file for instructions on installation and use. Also included in the distribution is a collection of UIMA wrappers for some commonly used BioNLP tools and annotated corpora. [file 1747-5333-3-1-S1.zip › bionlp_uima_repository-1.0.1/docs/api/edu/uchsc/ccp/util/external/abner/Abner_Util.html]

Abner\_Util (Center for Computational Pharmacology Util/UIMA API)


|  |  |  |
| --- | --- | --- |
| **Overview** \| **Package** \| **Class** \| **Tree** \| **Deprecated** \| **Index** \| **Help** | |  |
| PREV CLASS   NEXT CLASS | **FRAMES**    **NO FRAMES**     **All Classes** |  |
| SUMMARY: NESTED \| FIELD \| CONSTR \| METHOD | DETAIL: FIELD \| CONSTR \| METHOD |  |


---


## edu.uchsc.ccp.util.external.abner Class Abner\_Util

```
java.lang.Object
  edu.uchsc.ccp.util.external.abner.Abner_Util
```

---

``` public class Abner_Util extends Object ```

crap

This is an interface to Burr Settles' named entity recognizing application, ABNER.   
http://pages.cs.wisc.edu/~bsettles/abner/

Currently, this interface allows the user to specify the two models that come prepackaged in the abner.jar file. By default the ABNER tokenization
routine is used.

Extracted entities are returned as instances of `edu.uchsc.ccp.util.nlp.annotation.TextAnnotation`.

When the built-in BioCreative model is used, only protein annotations are returned. When the NLPBA model is used, protein, RNA, DNA, cell line, and
cell type annotations are returned.

**Author:**
:   William A. Baumgartner Jr.

---

**Constructor Summary**

`Abner_Util(int model)`
  
          Create a new Abner\_Util.


| **Method Summary** | |
| --- | --- |
| `List<TextAnnotation>` | `getEntities(String text, int documentCollectionID, String documentID)`             Retrieve the entities extracted by ABNER from text as instances of `edu.uchsc.ccp.util.nlp.annotation.TextAnnotation`. |

**Methods inherited from class java.lang.Object**

`clone, equals, finalize, getClass, hashCode, notify, notifyAll, toString, wait, wait, wait`

**Constructor Detail**

### Abner\_Util

```
public Abner_Util(int model)
```

:   Create a new Abner\_Util.

    **Parameters:**: `model` - The two default ABNER models can be selected. For the BioCreative model, use `tagger.BIOCREATIVE`. For the NLPBA model, use `tagger.NLPBA`.


**Method Detail**

### getEntities

```
public List<TextAnnotation> getEntities(String text,
                                        int documentCollectionID,
                                        String documentID)
```

:   Retrieve the entities extracted by ABNER from text as instances of `edu.uchsc.ccp.util.nlp.annotation.TextAnnotation`.

    :   **Parameters:**: `text` - The text to search for entities.: `documentCollectionID` - This unique identifier for a document collection will be assigned to any annotations returned by this method.: `documentID` - This unqiue identifier for a document will be assigned to any annotations returned by this method. **Returns:**: a List of `edu.uchsc.ccp.util.nlp.annotation.TextAnnotation` instances, one per entity detected in the text.


---


|  |  |  |
| --- | --- | --- |
| **Overview** \| **Package** \| **Class** \| **Tree** \| **Deprecated** \| **Index** \| **Help** | |  |
| PREV CLASS   NEXT CLASS | **FRAMES**    **NO FRAMES**     **All Classes** |  |
| SUMMARY: NESTED \| FIELD \| CONSTR \| METHOD | DETAIL: FIELD \| CONSTR \| METHOD |  |


---
